# Partial Failure of Proteostasis Systems Counteracting TDP-43 Aggregates in Neurodegenerative Diseases

**DOI:** 10.3390/ijms20153685

**Published:** 2019-07-27

**Authors:** Roberta Cascella, Giulia Fani, Alessandra Bigi, Fabrizio Chiti, Cristina Cecchi

**Affiliations:** Department of Experimental and Clinical Biomedical Sciences, University of Florence, V.le G.B. Morgagni 50, 50134 Florence, Italy

**Keywords:** autophagy, proteasome, FTLD, ALS, protein aggregation, calcium dyshomeostasis

## Abstract

Frontotemporal lobar degeneration (FTLD) and amyotrophic lateral sclerosis (ALS) are progressive and fatal neurodegenerative disorders showing mislocalization and cytosolic accumulation of TDP-43 inclusions in the central nervous system. The decrease in the efficiency of the clearance systems in aging, as well as the presence of genetic mutations of proteins associated with cellular proteostasis in the familial forms of TDP-43 proteinopathies, suggest that a failure of these protein degradation systems is a key factor in the aetiology of TDP-43 associated disorders. Here we show that the internalization of human pre-formed TDP-43 aggregates in the murine neuroblastoma N2a cells promptly resulted in their ubiquitination and hyperphosphorylation by endogenous machineries, mimicking the post-translational modifications observed in patients. Moreover, our data identify mitochondria as the main responsible sites for the alteration of calcium homeostasis induced by TDP-43 aggregates, which, in turn, stimulates an increase in reactive oxygen species and, finally, caspase activation. The inhibition of TDP-43 proteostasis in the presence of selective inhibitors against the proteasome and macroautophagy systems revealed that these two systems are both severely involved in TDP-43 accumulation and have a strong influence on each other in neurodegenerative disorders associated with TDP-43.

## 1. Introduction

Frontotemporal lobar degeneration with ubiquitin positive inclusions (FTLD-U) and amyotrophic lateral sclerosis (ALS) are devastating neurodegenerative diseases characterized by the mislocalization and cytosolic accumulation of the predominately nuclear TAR DNA-binding protein 43 (TDP-43) in the central nervous system [1,2]. FTLD-U is a cortical dementia, involving degeneration of the frontal and temporal brain lobes and hippocampus, characterized by TDP-43 positive inclusions in the neurons [1]. By contrast, in ALS patients, the inclusions bodies of TDP-43 were mainly found to accumulate in the upper and lower motoneurons of the brain, brainstem, and spinal cord, causing a progressive degeneration of these cells [3]. The presence of TDP-43 neuropathology in ~97% of ALS and ~50% of FTLD cases [4] provides a molecular link, connecting both diseases as a clinicopathological spectrum of the TDP-43 proteinopathies [1,5,6].

A number of physiological functions are perturbed in FTLD and ALS, including impaired protein homeostasis, RNA dysmetabolism, and reduced nucleocytoplasmic transport of mRNAs and proteins [4,7,8]. The cytoplasmic deposition of TDP-43 occurs concomitantly with the depletion of native TDP-43 from the nucleus [1], causing neurodegeneration in both FTLD-U and ALS by a combination of gain-of-function (GOF) and loss-of-function (LOF) mechanisms [1,9,10]. Cytosolic aggregates are known to be intrinsically toxic [10,11] and able to recruit nuclear TDP-43, exacerbating their deleterious effects by contributing to the nuclear LOF [12,13,14]. Recent studies indicate that Ca^2+^ disturbances, endoplasmic reticulum (ER) stress, and mitochondrial dysfunction are involved in the pathogenesis of both diseases [15,16,17]. Other findings also indicate that TDP-43 is capable of inducing neurodegeneration due to the compromised function of the chromatin remodeller Chd1/CHD2, that prevents the appropriate expression of protective genes [18].

In this scenario, it is crucial for neurons to maintain TDP-43 protein homeostasis by the ubiquitin-proteasome system (UPS) and the autophagy-lysosomal pathway (ALP) [19,20,21,22]. Indeed, a progressive decrease in the efficiency of both protein degradation systems has been reported as a major factor contributing to FTLD-U and ALS onset in aging patients. This hypothesis is also supported by genetic evidence, as many of the mutations associated with FTLD and ALS affect genes involved in UPS- or ALP-mediated degradation, such as VCP, CHMP2B, OPTN, SQSTM1, TBK1, UBQLN2, FIG4, ALS2, and C9ORF72 [23,24,25,26,27,28,29,30,31,32,33,34,35]. Recent studies showed that the cytosolic accumulation of TDP-43 is turned-over mainly by the UPS, with ALP contributing to its degradation when TDP-43 accumulates as intractable aggregates [21,36]. Accordingly, we recently found that cytosolic diffuse TDP-43 can be assembled in different species of misfolded TDP-43, ranging from soluble monomers to undegradable macroaggregates [36].

Here we found an early ROS production and caspase-3 activation in murine neuroblastoma N2A cells, following cytosolic transfection with preformed inclusions of human TDP-43 in the absence of any recruitment of the nuclear TDP-43 reservoir. Moreover, our findings identify the mitochondria as the main responsible organelle for the alteration of calcium homeostasis induced by TDP-43 aggregates. We also showed that the UPS and ALP systems are unable to efficiently degrade at least a fraction of the TDP-43 inclusions.

## 2. Results

### 2.1. TDP-43 Aggregates Are Ubiquitinated and Phosphorylated in Neuronal Cells

**Figure 1:**
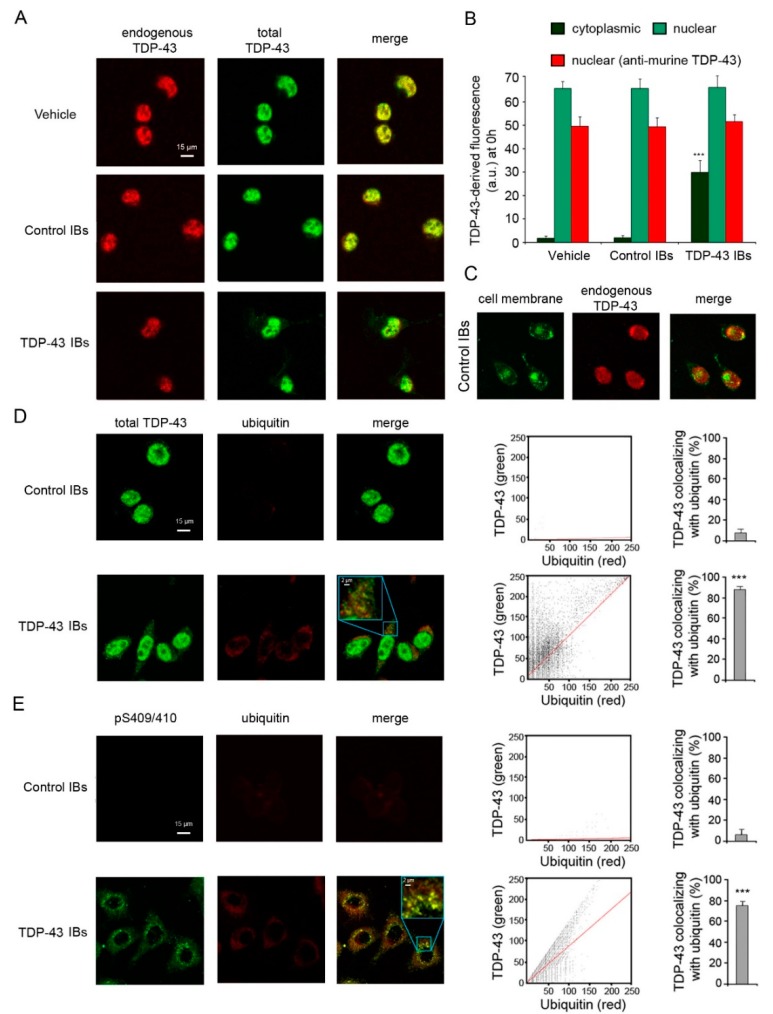
TDP-43 inclusions are ubiquitinated and phosphorylated in N2A neuroblastoma cells. (**A**) Representative confocal scanning microscope images of N2A cells transfected with vehicle, control IBs, and TDP-43 IBs. Red and green fluorescence indicate endogenous murine TDP-43 and total TDP-43 (human and murine), respectively, detected with immunofluorescence. (**B**) Quantitative analysis of cytoplasmic (green bars) and nuclear (pale green bars) TDP-43-derived fluorescence 0 h after transfection and using antibodies specific for total (human and murine) TDP-43. The red bars refer to nuclear TDP-43-derived fluorescence at 0 h using antibodies specific for murine TDP-43. (**C**) Representative confocal scanning microscope images of N2A cells transfected with control IBs. Green and red fluorescence indicate cell membrane and endogenous murine TDP-43, respectively. (**D**,**E**) Representative confocal scanning microscope images showing the co-localization of cytoplasmic TDP-43 (**D**) or TDP-43 phosphorylated at Ser409/410 (**E**) with ubiquitin-positive aggregates in N2A cells transfected with control IBs or TDP-43 IBs. Green and red fluorescence indicate total TDP-43 or pS409/410 TDP-43 and ubiquitin, respectively. Higher magnifications of the merged images are shown in the boxed areas. The cytofluorograms show the cytoplasmic green fluorescence intensity (as pixel intensity, y axis) versus cytoplasmic red fluorescence intensity (x axis). The histograms show the percentage of co-localization on regions of interest (50–60 cells) using the ImageJ and JACOP plugin software. Error bars are S.D. *** *p* < 0.001 relative to cells transfected with vehicle.

TDP-43 inclusion bodies (TDP-43 IBs) and control inclusion bodies (control IBs) were obtained from *E. coli* expressing the human form of TDP-43 or an empty vector, respectively, tagged with the GST. When TDP-43 inclusions were internalized into murine N2A neuroblastoma cells using lipofectamine, a dense nuclear localization and a diffuse cytosolic distribution of TDP-43 (green channel) were shown by scanning confocal microscopy using monoclonal antibodies that recognize both murine and human isoforms of TDP-43 protein (Figure 1A,B). In contrast, just a nuclear localization of the endogenous TDP-43 protein (red channel) was evident in cells transfected with TDP-43 IBs using antibodies specific for the murine isoform of TDP-43 (Figure 1A,B). The cells transfected with vehicle or control IBs showed only a nuclear TDP-43 fluorescence (Figure 1A–C). Of importance, the levels of nuclear endogenous TDP-43 at 0 h were similar in the various conditions (Figure 1B), indicating that the transfection procedure did not change the levels of nuclear endogenous TDP-43.

It is well established that the formation of cytoplasmic TDP-43 inclusions in neurons of FTLD-U and motoneurons of ALS patients is accompanied by post-translational modifications of the TDP-43 protein, including ubiquitination and hyperphosphorylation [1]. Here, the images obtained by scanning confocal microscopy showed a highly significant co-localization between exogenous cytosolic TDP-43 inclusions and ubiquitin (~85%) in N2A neuroblastoma cells, with respect to cells transfected with control IBs (Figure 1D). The cytosolic TDP-43 inclusions also appeared phosphorylated at S409/410 sites, with a very significant co-localization with ubiquitin (~76%) (Figure 1E). By contrast, a negligible phosphorylation and ubiquitin staining was evident in the cytoplasm of N2A cells transfected with control IBs (Figure 1E). These data suggest that ubiquitinated and phosphorylated inclusions of the transfected human TDP-43 are localized in the cytosol of our neuronal model, recapitulating the post-translational modifications observed in neurodegenerative disorders.

### 2.2. TDP-43 Aggregates Cause Ca^2+^ Release from Mitochondrial Stores in Neuronal Cells

**Figure 2:**
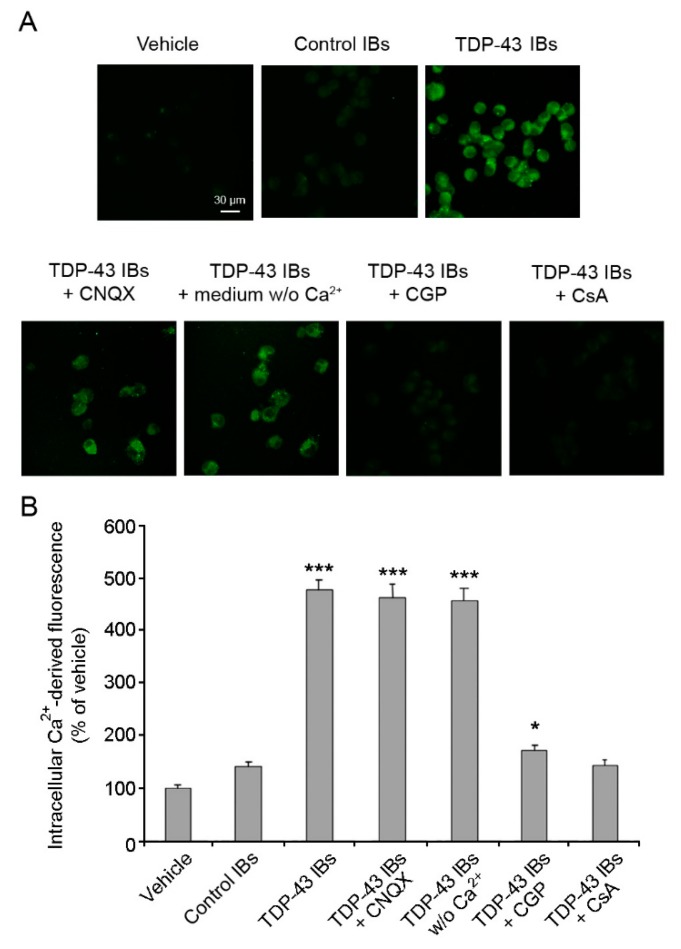
Analysis of intracellular Ca^2+^ levels. (**A**) Representative confocal scanning microscope images of N2A cells showing intracellular Ca^2+^ levels after transfection with vehicle, control IBs, and TDP-43 IBs in the absence or presence of 5 µM CNQX, 5 µM CGP, or 10 µM CsA or in medium without Ca^2+^. The green fluorescence arises from the intracellular Fluo4 probe bound to Ca^2+^. (**B**) Quantitative analysis of the green fluorescence arising from the Fluo4 probe. Error bars are S.D. * *p* < 0.05 and *** *p* < 0.001 relative to cells transfected with vehicle.

Several studies have shown abnormalities of Ca^2+^ homeostasis in both FTLD and ALS [7,8,37,38]. By using confocal microscopy and Fluo4 as a specific fluorescent probe, we found that transfected TDP-43 aggregates triggered a significant and extensive increase of Ca^2+^ levels in the cytosol of N2A neuroblastoma cells (Figure 2). On the contrary, cells treated with control IBs showed a cytosolic Ca^2+^ content that was unchanged compared to cells treated with lipofectamine (vehicle). Then, we investigated whether calcium dyshomeostasis was caused by a destabilization of the calcium channels on the plasma membrane or by an imbalance of the mitochondrial calcium uptake. Thus, before TDP-43 IB internalization we incubated the cells with 5.0 μM 6-cyano-7-nitroquinoxaline-2,3-dione (CNQX), which is a competitive antagonist of α-amino-3-hydroxy-5-methyl-4-isoxazolepropionic acid receptors (AMPA-R), or with 5 µM CGP-37157 (CGP), which is an inhibitor of the mitochondrial Na^+^/Ca^2+^ exchanger, or with 10 µM cyclosporine A (CsA), which is an inhibitor of the mitochondrial permeability transition pore (mPTP). Pre-treatment with CNQX did not prevent the alteration of Ca^2+^ homeostasis induced by TDP-43 inclusions, ruling out the involvement of the AMPA receptor in intracellular Ca^2+^ dyshomeostasis (Figure 2). Similar results were obtained in cells treated with TDP-43 inclusions in the presence of a cell medium without Ca^2+^, ruling out that the increase of intracellular Ca^2+^ levels had an extracellular origin. In contrast, both CGP and CsA inhibitors significantly reduced the intracellular Ca^2+^ increase induced by TDP-43 inclusions, indicating the involvement of mitochondria in the disruption of Ca^2+^ homeostasis (Figure 2).

**Figure 3:**
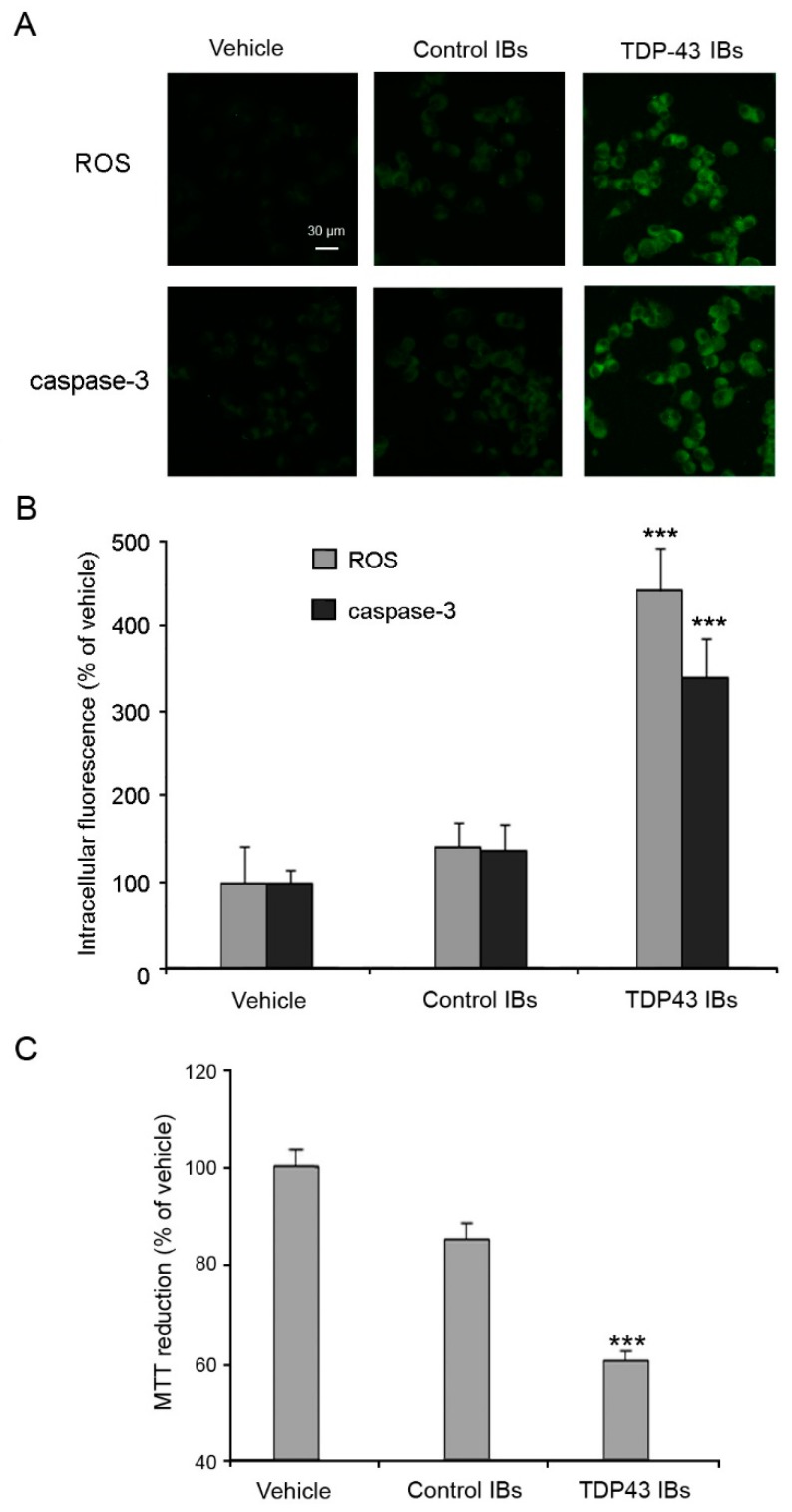
Analyses of intracellular ROS production, caspase-3 activation, and cell viability. (**A**) Representative confocal scanning microscope images of N2A cells showing intracellular ROS production and caspase-3 activation after transfection with vehicle, control IBs, and TDP-43 IBs. The green fluorescence arises from the CM-H_2_DCFDA probe that has reacted with ROS and from FAM-FLICA^TM^ Caspase 3&7, respectively. (**B**) Quantitative analysis of the green fluorescence arising from CM-H_2_DCFDA and FAM-FLICA^TM^ Caspase 3&7 probes. (**C**) MTT reduction of N2a cells transfected with vehicle, control IBs and, TDP-43 IBs and analyzed after 24 h. Error bars are S.D. *** *p* < 0.001 relative to cells transfected with vehicle.

Moreover, TDP-43 inclusions caused a significant increase in ROS production and caspase-3 activation in N2A neuronal cells (Figure 3A,B). In contrast, cells treated with lipofectamine (vehicle) or with control IBs showed a negligible alteration of the redox and apoptotic status (Figure 3A,B). Cells transfected with TDP-43 IBs also showed a significant decrease of cell viability with respect to cells treated with vehicle, as assessed by the MTT test (Figure 3C). On the contrary, control IBs showed a modest level of cytotoxicity, attributable to the aggregated protein present in the IBs. These findings show that the cytosolic accumulation of TDP-43 inclusions stimulates the release of calcium from the mitochondrial stores, inducing ROS production and activating the apoptotic pathway, thus summarizing the pathological hallmarks of FTLD and ALS.

### 2.3. TDP-43 Aggregates Are Degraded by Proteasome and Autophagy Systems

**Figure 4:**
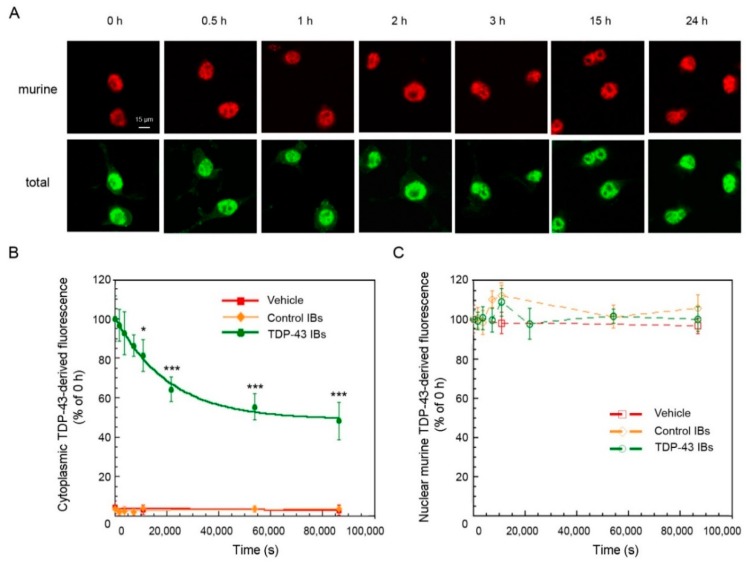
TDP-43 clearance in N2A following the transfection with TDP-43 aggregates. (**A**) Representative confocal scanning microscope images of N2A cells that were transfected with TDP-43 IBs and analyzed at the indicated time points after transfection. Red and green fluorescence indicates endogenous murine TDP-43 and total TDP-43 (human and murine) detected with immunofluorescence. (**B**,**C**) Kinetic plots showing cytoplasmic (**B**) and nuclear (**C**) TDP-43 derived fluorescence, detected with Abs against total (**B**) and only murine (**C**) TDP-43 as a function of time after transfection of N2A cells with vehicle (red), control IBs (yellow), and TDP-43 IBs (green). The line in panel (**B**) represents the best fits to an exponential function. Error bars: S.D. * *p* < 0.05 and *** *p* < 0.001 relative to cells analyzed 0 h after transfection.

The transfection of N2A cells with human TDP-43 inclusions allows for following the clearance of the TDP-43 aggregates present at time 0 h, independently of the occurrence of pre-existing degradation. We thus followed the levels of both nuclear and cytoplasmic TDP-43 in N2A cells from 0 h to 24 h after transfection, using both antibodies specific for the total (human and murine) and only the endogenous murine TDP-43 (Figure 4A). In cells treated with TDP-43 inclusions, the cytosolic levels of TDP-43 (green signal) decreased over time with apparent single exponential kinetics, whereas negligible levels of cytosolic endogenous TDP-43 (red signal) were found over the 24 h time period using anti-murine TDP-43 (Figure 4A,B). Accordingly, the nuclear levels of endogenous TDP-43 (red signal) were found to remain constant over the explored 24 h time period after transfection with TDP-43 IBs in N2A cells (Figure 4A,C), indicating that nuclear TDP-43 was not recruited to the cytosol by exogenous TDP-43 inclusions in our neuronal model. A plot of the cytosolic TDP-43 levels *versus* time showed an apparent rate constant of clearance of 4.78(±0.59) × 10^−5^ s^−1^, a 52.11(±2.16)% of initial cytoplasmic TDP-43 aggregates that have been degraded, and a 48.67(±2.02)% of undegradable TDP-43 at the end of the exponential phase (Figure 4B).

**Figure 5:**
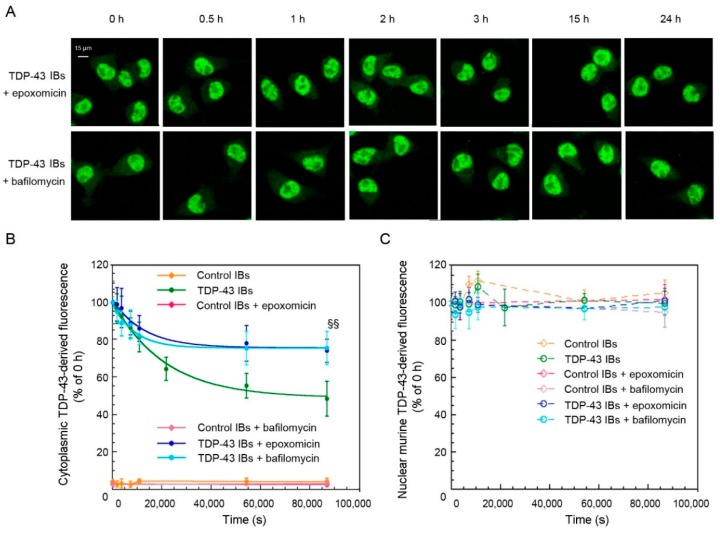
TDP-43 levels in N2A cells following the treatment with UPS and ALP inhibitors and the transfection with TDP-43 aggregates. (**A**) Representative confocal scanning microscope images of N2A cells that were treated or not with 100 nM epoxomicin or 400 nM bafilomycin, then transfected with TDP-43 IBs and analyzed at the indicated time points after transfection. Green fluorescence indicates total TDP-43 (human and murine) detected with immunofluorescence. (**B**,**C**) Kinetic plots showing cytoplasmic (**B**) and nuclear (**C**) TDP-43 derived fluorescence, detected with Abs, against the total TDP-43 as a function of time after transfection of N2A cells with control IBs in the absence (yellow) or in the presence of a pre-treatment with epoxomicin (magenta) or bafilomycin (pink) and TDP-43 IBs in the absence (green) or in the presence of a pre-treatment with epoxomicin (blue) or bafilomycin (cyan). The line in panel (**B**) represents the best fits to an exponential function. Error bars are S.D. ^§§^ *p* < 0.01 relative to cells without inhibitors.

To further study the efficiency of the clearance systems in N2A neurons after cytosolic insertion of TDP-43 inclusions, the cells were pre-treated for 8 h with either the UPS inhibitor epoxomicin or the ALP inhibitor bafilomycin and then the levels of TDP-43 were analyzed using specific antibodies (Figure 5). When TDP-43-derived fluorescence was plotted *versus* time, apparent single exponential kinetics were observed in all cases (Figure 5A,B). The 25.75(±2.05)% value of decreased fluorescence obtained in the presence of epoxomicin represents the fraction of aggregated cytoplasmic TDP-43 that is degradable via the ALP (Figure 5B), a percent level significantly lower (*p* < 0.001) than that observed in the absence of any inhibitor, which is 52.11(±2.16)%. A 23.91(±1.74)% value of decreased fluorescence was obtained in the presence of bafilomycin, which reports approximately on the fraction degradable via the UPS (Figure 5B), a value again significantly lower (*p* < 0.001) than that observed in the absence of inhibitors. This result indicates that both UPS and ALP contribute to the clearance of exogenous inserted TDP-43 aggregates. The same analysis was repeated using antibodies specific for only murine TDP-43, confirming that all the TDP-43 detected in the cytoplasm was exogenous (human) and that the levels of endogenous murine TDP-43 remained constant for the length of our time courses in the presence of the inhibitors (Figure 5C). In the presence of epoxomicin or bafilomycin, the apparent rate constants of clearance were found to be 7.84(±1.68) × 10^−5^ s^−1^ and 11.37(±1.94) × 10^−5^ s^−1^, respectively, higher than that obtained in the absence of inhibitors, which is 4.78(±0.59) × 10^−5^ s^−1^ (*p* < 0.05 and *p* < 0.001, respectively). This implies that when either the UPS or ALP is inhibited, the active clearance system works more rapidly, albeit the extent of disposed aggregated TDP-43 is eventually lower. As a negative control, we also monitored the levels of exogenous and endogenous TDP-43 in cells pre-treated with epoxomicin or bafilomycin and transfected with control IBs (Figure 5B,C).

## 3. Discussion

The decrease in the efficiency of the clearance systems in aging, as well as the presence of gene mutations associated with cellular proteostasis in the familial forms of TDP-43 proteinopathies, suggests that a failure of these protein degradation systems is a major factor in the aetiology of TDP-43 associated disorders [39,40,41]. In this work we have transfected murine neuroblastoma N2A cells with human TDP-43 aggregates and followed their ability to cause cell dysfunction along with the mechanism of their clearance. Different methodologies were previously used to induce the accumulation of TDP-43 in the cytoplasm, either by inducible gene expression or pre-formed aggregates transfection in different cell models, such as human, SH-SY5Y and HEK293, or murine, NSC34 and N2a. Several techniques were also used to analyze the TDP-43 species accumulating in the cytosol, such as immunohistochemistry and FRAP [21,36]. Notably, the TDP-43 inclusions internalized in N2A neuroblastoma cells were found to be ubiquitinated and phosphorylated at Ser409/410 residues, recapitulating the post-translational modifications observed in neurodegenerative disorders [1,5,6,12]. 

The link between mitochondria and TDP-43 proteinopathies has been well established in patients affected by both sporadic and familiar forms of these diseases [42]. Intracellular calcium shuttles between the ER and mitochondria [43] and has been found to mediate apoptosis and other forms of cell death [44]. In this study, the cytosolic TDP-43 inclusions were found to stimulate the release of calcium from the mitochondrial stores, inducing ROS production and activating the apoptotic pathway, thus causing a significant reduction of cell viability. TDP-43 was previously shown to colocalize with mitochondrial markers in cellular and animal models of ALS and, thus, might alter the functions of these organelles [45]. Since oxidative phosphorylation in the mitochondria is the major source of ROS, oxidative stress may be linked to abnormalities in these organelles.

It has previously been established that both the UPS and ALP contribute to the clearance of cytosolic TDP-43 in both cellular and animal models [19,20,21,36]. Our analysis has shown that, under conditions in which TDP-43 aggregates are present, both the UPS and ALP contribute to their degradation and that a pool of TDP-43 inclusions cannot be degraded, confirming previous findings [21,36]. Among the three TDP-43 aggregates distinguishable here on the basis of their susceptibility to the various forms of protein degradation, the un-disposable fraction corresponds to ca. 49%, whereas those degradable via UPS and ALP account for ca. 24% and 26%, respectively, indicating that both routes contribute equally to degradation of pre-formed TDP-43 assemblies. However, the UPS- and ALP-mediated degradation processes appear to be kinetically independent, acting on different populations of TDP-43 aggregates that are not in equilibrium or that interconvert on a slow time scale. Indeed, the inhibition of one system would cause a deceleration of the degradation process and would result in similar levels of degraded TDP-43, if the two populations were at equilibrium. By contrast, the rate constant of degradation is significantly higher and the amount of degraded TDP-43 is lower when either UPS or ALP is inhibited relative to the condition in which both routes are at work. This finding confirms previous observations that the inhibition of one clearance pathway renders the remaining one more effective [46,47] and reinforces the view that a cross-talk exists between the two clearance systems. These findings are in agreement with our recent results obtained in NSC34 motor neurons [36], confirming a common molecular mechanism in different cell types endowed with different UPS and ALP equipment. Our evidence also suggests the importance of extending the analysis to future in vivo murine models in order to test selective modulators of the UPS and ALP routes as effective drugs for the therapy of neurodegenerative diseases with TDP-43 proteinopathies.

## 4. Materials and Methods

### 4.1. Purification of TDP-43 Inclusions and Internalization in N2a Neuroblastoma Cells

The gene for human full-length TDP-43 (residues 1–414) was cloned downstream of the glutathione S-transferase (GST) gene in the pGEX-2T plasmid. Cultures of *E. coli* XL1 Blue cells (Agilent Technologies, Santa Clara, CA, USA) were transformed with the resulting plasmid. Inclusion bodies were purified from IPTG induced bacteria harboring the pGEX-2T/TDP-43 plasmid (TDP-43 IBs) and the only pGEX-2T plasmid (control IBs) by detergent-based procedures, as previously described [14]. Authenticated murine Neuro2a (N2a) neuroblastoma cells (A.T.C.C., Manassas, VA, USA) were cultured in minimum essential medium eagle (MEM), supplemented with 10% FBS, 1 mM glutamine, and 1% antibiotics. Cell cultures were maintained in a 5% CO_2_ humidified atmosphere at 37 °C and grown until they reached 80% confluence for a maximum of 20 passages. The cells were tested negatively for mycoplasma contaminations. Cells were plated in 6-well plates containing coverslips at 30,000 cells/well density [13,48]. The day after, the cells were transfected for 2 h using 4 μL of the PULSin protein delivery reagent (Polyplus-transfection, Illkirch, France), without IBs (vehicle), with control IBs (4 μg/mL final concentration), or with TDP-43 IBs (5.7 μg/mL final concentration) in culture medium without FBS, according to the manufacturer’s instructions. In all our analyses, we used concentrations of TDP-43 IBs that were 30% higher than control IBs so that the two sets of IBs differed only in the presence of TDP-43 aggregates [14]. After transfection, the culture medium was replaced with fresh MEM with 10% FBS and the biological analyses were performed.

### 4.2. Confocal Microscopy Analysis of TDP-43 Inclusions in N2A Neuroblastoma Cells

The levels of TDP-43 were analyzed following the transfection of TDP-43 IBs or control IBs or vehicle to N2A cells seeded on glass coverslips. After transfection, N2a cells were washed with PBS, fixed in 2% (*w*/*v*) buffered paraformaldehyde for 10 min at room temperature (20 °C), and permeabilized with a 0.5% (*v*/*v*) Triton X-100 solution for 5 min. Then the cells were incubated for 60 min at 37 °C with 1:500 mouse monoclonal anti-TDP-43 antibodies (Novus Biologicals, Littleton, CO, USA) or with 1:500 rabbit polyclonal anti-murine TDP-43 antibodies (LSBio, Seattle, WA, USA), that only recognize the murine endogenous protein, and then for 90 min with 1:1000 diluted Alexa Fluor 488-conjugated anti-mouse or 594-conjugated anti-rabbit secondary antibodies, respectively, as previously reported [21,36]. In a set of experiments, following the transfection with control IBs, the cells were counterstained for 15 min with 5.0 μg/mL Alexa Fluor 488-conjugated wheat germ agglutinin (Thermo Fisher Scientific, Waltham, MA, USA) and fixed in 2% (*w*/*v*) buffered paraformaldehyde for 10 min at room temperature (20 °C). After plasma membrane permeabilization with a 0.5% (*v*/*v*) Triton X-100 solution for 5 min, the coverslips were incubated for 60 min with 1:500 rabbit polyclonal anti-murine TDP-43 antibodies (LSBio, Seattle, WA, USA) and then for 90 min with 1:1000 diluted Alexa Fluor 594-conjugated anti-rabbit antibodies. Cells were analyzed after double excitation at 488 and 594 nm using a Leica TCS SP5 confocal scanning microscope (Leica Microsystems, Mannheim, Germany) equipped with an argon laser source and a Leica Plan Apo 63× oil immersion objective. A series of optical sections (1024 × 1024 pixels), 1.0 μm in thickness, was taken through the cell depth for each examined sample. Between 50–60 cells were analyzed on regions of interest (ROI) for each examined sample, in three different experiments, using the ImageJ software (NIH, Bethesda, MD, USA). Cytosolic TDP-43 levels (green) were assessed by selecting multiple ROIs per cell in the cytoplasmic compartment, excluding the nucleus area, following background subtraction. Similarly, nuclear TDP-43 levels (red) were measured by selecting one–two ROIs per cell in the nuclear compartment, excluding the cytoplasmic area, following the background subtraction. The fluorescence intensities were expressed as percentages of cells analyzed immediately after the 2 h transfection phase (time 0 h), taken as 100%.

### 4.3. Analysis of Phosphorylation and Ubiquitination of TDP-43 IBs

The analysis of phosphorylation and ubiquitination of TDP-43 was carried out following the transfection of TDP-43 IBs or control IBs to N2A cells seeded on glass coverslips (30,000 cells/well density). After washing with PBS, cells were fixed in 2% (*w*/*v*) buffered paraformaldehyde for 10 min at room temperature (20 °C) and permeabilized with a 0.5% (*v*/*v*) Triton X-100 solution for 5 min. The co-localization of TDP-43 or phosphorylated TDP-43 with ubiquitin was monitored using 1:350 rabbit polyclonal anti-TDP-43 antibody (Sigma-Aldrich, St. Louis, MO, USA) or 1:500 rabbit anti-TDP-43 phosphorylation sites 409/410 (Cosmo Bio Co., Ltd., Tokyo, Japan) for 60 min at 37 °C, 1:150 mouse monoclonal anti-ubiquitin antibodies (Thermo Fisher Scientific) for 60 min at 37 °C, and then with 1:1000 Alexa Fluor 488- and 594-conjugated secondary antibodies (Thermo Fisher Scientific) for 60 min at 37 °C, as previously described [13]. The co-localization of TDP-43 with ubiquitin was estimated for the regions of interest (50–60 cells) in three different experiments using the ImageJ (NIH, Bethesda, MD, USA) and JACOP plugin software (Rasband, 1997–2008, rsb.info.nih.gov).

### 4.4. Analysis of Ca^2+^ Levels

The cytosolic levels of free Ca^2+^ were measured following the transfection of TDP-43 or control inclusions to N2A cells seeded on glass coverslips and loaded with 4.0 µM Fluo4-AM (Thermo Fisher Scientific), as previously described [49]. In a set of experiments, cells were pre-treated for 60 min with 5.0 µM 6-cyano-7-nitroquinoxaline-2,3-dione (CNQX), a competitive antagonist of α-amino-3-hydroxy-5-methyl-4-isoxazolepropionic acid receptors (AMPA-R) [49,50], with 5 µM CGP-37157 (CGP) (Sigma-Aldrich, St. Louis, MO, USA), an inhibitor of the mitochondrial Na^+^/Ca^2+^ exchanger [51], or with cyclosporine A (CsA) (Sigma-Aldrich), an inhibitor of the mitochondrial permeability transition pore (mPTP) [52], or with culture medium without Ca^2+^. Fluorescence emission was detected after excitation at 488 nm by using the confocal microscope described above. To quantify the fluorescent signal intensities, 80–100 cells were analyzed using ImageJ software (NIH, Bethesda, MD, USA) and the fluorescence intensities were expressed as percentages of cells treated with vehicle, taken as 100%.

### 4.5. Measurement of Intracellular ROS Production, Caspase-3 Activation and Cell Viability

Intracellular ROS production and caspase-3 activity were analyzed following the transfection of TDP-43 or control inclusions to N2A cells seeded on glass coverslips and loaded with CM-H_2_DCFDA and using FAMFLICA™ Caspases 3&7 solution (Caspase 3&7 FLICA kit FAM-DEVDFMK, Immunochemistry Technologies, LLC, Bloomington, MN, USA), as previously reported [13,14]. Fluorescence emission was detected after excitation at 488 nm by using the confocal microscopy described above. To quantify the fluorescent signal intensities, 80–100 cells were analyzed using ImageJ software (NIH, Bethesda, MD, USA), and the fluorescence intensities were expressed as percentages of cells treated with vehicle, taken as 100%.

The cytotoxicity of TDP-43 inclusions was assessed on N2A cells seeded in 96-well plates, 24 h following the transfection of TDP-43 IBs or control IBs, by the 3-(4,5-dimethylthiazol-2-yl)-2,5-diphenyltetrazolium bromide (MTT) assay, as previously described [13]. Cell viability was expressed as the percentage of MTT reduction relative to cells that were treated with vehicle, taken as 100%.

### 4.6. Time-Course of TDP-43 Degradation in the Absence or Presence of UPS and ALP Inhibitors

TDP-43 degradation was analyzed in N2a cells plated in 6-well plates (30,000 cells/well density) at 0 min, 30 min, 1 h, 2 h, 3 h, 15 h, and 24 h after transfection. In a set of experiments, the cells were pre-treated with 100 nM epoxomicin (Abcam, Cambridge, UK), a potent proteasome inhibitor [53], or with 400 nM bafilomycin (Abcam, Cambridge, UK), which inhibits autophagic flux by preventing the acidification of endosomes and lysosomes [54]. Then, the cells were washed with PBS, fixed in 2% (*w*/*v*) buffered paraformaldehyde for 10 min at room temperature (20 °C), and permeabilized with a 0.5% (*v*/*v*) Triton X-100 solution for 5 min. Then, the cells were incubated for 60 min at 37 °C with 1:500 mouse monoclonal anti-TDP-43 antibodies (Novus Biologicals, Littleton, CO, USA) or with 1:500 rabbit polyclonal anti-murine TDP-43 antibodies (LSBio, Seattle, WA, USA), which only recognize the murine protein, and for 90 min with 1:1000 diluted Alexa Fluor 488-conjugated anti-mouse or 594-conjugated anti-rabbit secondary antibodies, respectively, and then analyzed by confocal microscopy, as described above.

### 4.7. Statistical Analysis

Data were expressed as means ± standard deviation (S.D.). Comparisons between different groups were performed using one-way ANOVA followed by Bonferroni’s post-comparison test. A *p*-value lower than 0.05 was considered statistically significant.

## 5. Conclusion

In summary, these findings identify the mitochondria as the main responsible organelle for the early neuronal dysfunction induced by TDP-43 aggregates. Moreover, our evidences indicate a cross-talk between the UPS and ALP clearance systems, which appear to be unable to completely degrade the TDP-43 inclusions in neurodegenerative diseases.

## Abbreviations

ALP	autophagy-lysosomal pathway
ALS	Amyotrophic lateral sclerosis
CGP	CGP-37157
CNQX	6-cyano-7-nitroquinoxaline-2,3-dione
CsA	cyclosporine A
DMEM	Dulbecco’s Modified Eagle’s Medium
FBS	fetal bovine serum
FTLD	frontotemporal lobar degeneration
GST	glutathione S-transferase
MTT	3-(4,5-dimethylthiazol-2-yl)-2,5-diphenyltetrazolium bromide
TDP-43	TAR DNA-binding protein 43
TDP-43 IBs	TDP-43 inclusion bodies
UPS	ubiquitin-proteasome system
